# Hyaluronan-Cholesterol Nanogels for the Enhancement of the Ocular Delivery of Therapeutics

**DOI:** 10.3390/pharmaceutics13111781

**Published:** 2021-10-25

**Authors:** Nicole Zoratto, Laura Forcina, Roberto Matassa, Luciana Mosca, Giuseppe Familiari, Antonio Musarò, Maurizio Mattei, Tommasina Coviello, Chiara Di Meo, Pietro Matricardi

**Affiliations:** 1 Department of Drug Chemistry and Technologies, Sapienza University of Rome, 00185 Roma, Italy; nicole.zoratto@uniroma1.it (N.Z.); tommasina.coviello@uniroma1.it (T.C.); chiara.dimeo@uniroma1.it (C.D.M.); 2 DAHFMO-Unit of Histology and Medical Embryology, Sapienza University of Rome, Via A. Scarpa, 14, 00161 Rome, Italy; laura.forcina@uniroma1.it (L.F.); antonio.musaro@uniroma1.it (A.M.); 3 Department of Anatomical, Histological, Forensic and Orthopaedic Sciences, Section of Human Anatomy, Sapienza University of Rome, Via A. Borelli 50, 00161 Rome, Italy; roberto.matassa@uniroma1.it (R.M.); giuseppe.familiari@uniroma1.it (G.F.); 4 Department of Biochemical Sciences “A. Rossi Fanelli”, Sapienza University of Rome, 00185 Roma, Italy; luciana.mosca@uniroma1.it; 5 Interdepartmental Center for Comparative Medicine, Alternative Techniques and Aquaculture (CIMETA), University of Rome “Tor Vergata”, Via Montpellier 1, 00133 Rome, Italy; maurizio.mattei@uniroma2.it; 6 Department of Biology, University of Rome “Tor Vergata”, Via della Ricerca Scientifica 1, 00133 Rome, Italy

**Keywords:** hyaluronan, nanogels, ocular delivery, permeation enhancer

## Abstract

The anatomy and physiology of the eye strongly limit the bioavailability of locally administered drugs. The entrapment of therapeutics into nanocarriers represents an effective strategy for the topical treatment of several ocular disorders, as they may protect the embedded molecules, enabling drug residence on the ocular surface and/or its penetration into different ocular compartments. The present work shows the activity of hyaluronan-cholesterol nanogels (NHs) as ocular permeation enhancers. Thanks to their bioadhesive properties, NHs firmly interact with the superficial corneal epithelium, without penetrating the stroma, thus modifying the transcorneal penetration of loaded therapeutics. *Ex vivo* transcorneal permeation experiments show that the permeation of hydrophilic drugs (i.e., tobramycin and diclofenac sodium salt), loaded in NHs, is significantly enhanced when compared to the free drug solutions. On the other side, the permeation of hydrophobic drugs (i.e., dexamethasone and piroxicam) is strongly dependent on the water solubility of the entrapped molecules. The obtained results suggest that NHs formulations can improve the ocular bioavailability of the instilled drugs by increasing their preocular retention time (hydrophobic drugs) or facilitating their permeation (hydrophilic drugs), thus opening the route for the application of HA-based NHs in the treatment of both anterior and posterior eye segment diseases.

## 1. Introduction

Topical drug delivery is an easy, self-administrable and non-invasive procedure widely used to treat both anterior (e.g., dry eye disease or infections) and posterior (e.g., non-infectious posterior uveitis, diabetic retinopathy, retinal vein occlusion or age-related macular degeneration with macular oedema) segment diseases affecting the ocular tissues [1,2]. Among the different dosage forms, eye drops are the most common method for ocular drug administration [3]. Although topical instillation is characterised by a relatively higher patient acceptability than other routes (i.e., intracameral injection), its application still faces several challenges due to the anatomical and physiological barriers of the eye, which hinder drug penetration [4]. Hence, less than 5% of topically applied therapeutics reach the intraocular tissues [5]. Similarly, systemic administration of drugs (e.g., corticosteroids) usually requires high doses to achieve therapeutic drug levels in the eye, which quite often is accompanied by significant systemic side effects [6].

When a drug is topically administered, the cornea represents the main mechanical and chemical barrier limiting its diffusion into the anterior camera of the eye. Actually, the epithelium, the outer layer of the cornea, represents around 90% of resistance for hydrophilic drugs and 10% for hydrophobic ones [7]. On the opposite end, the lower hydrophilic layer, stroma, acts to constrain very hydrophobic molecules, whilst the inner endothelium provides only a small resistance to ocular drug delivery [8]. Such a sandwich-like structure prevents the permeation of hydrophilic and most hydrophobic drugs [9]. Thus, transcorneal drug permeation is significantly affected by the physico-chemical properties of the bioactive molecules, mainly by the molecular weight, the lipophilicity (log D) and the drug charge density, as the corneal surface exhibits a net negative charge under physiological conditions (isoelectric point, pI = 3.2) [9,10]. In particular, small hydrophobic drugs can cross corneal epithelium through transcellular pathways and accumulate there, whilst the stroma may act as a reservoir from which such compounds are slowly delivered to the inner ocular tissue [11]. On the other side, the hydrophilic drugs may cross the cornea by the paracellular route which is hindered by the tight junctions of the corneal epithelial cells, thus preventing the corneal permeation of most of these molecules [12]. Thus, only small lipophilic molecules (log D = 2–3) and cationic drugs can effectively permeate the cornea [9,13].

For the above-described reasons, nanocarriers able to efficiently load therapeutics and interact with the ocular surface components represent an appealing platform for facilitating drug permeation across these barriers and enhancing ocular drug delivery [14]. A wide range of natural and synthetic polymeric nanogels (NHs) have been formulated to improve the bioavailability of ophthalmic drugs [15,16,17,18]. NHs composed of natural polymers, e.g., polysaccharides, show the additional advantage of being usually mucoadhesive, non-toxic, biocompatible and biodegradable [19]. Among the natural polymers, hyaluronic acid or hyaluronan (HA), a linear polysaccharide made up of repeating units of N-acetyl-d-glucosamine and d-glucuronic acid, linked together via alternating β-1,3 and β-1,4 glycosidic bonds, represents a good candidate for the preparation of NHs [20]. HA naturally occurs in the human body, and the highest concentrations can be found in the joints and in the eyes, where it is located in both corneal endothelium and epithelium, thanks to its interaction with the CD44 receptors of cells. Enhanced expression of CD44 was observed on the epithelium of corneas with inflammation and allograft rejection. Moreover, a number of pathological conditions which affect the cornea, including corneal trauma, primary and secondary corneal endothelial decompensation and allograft rejection, result in alternative splicing of the CD44 isotype and an increase in receptor density [21]. As a result, HA is widely used in ophthalmology during eye surgery and for the treatment of dry eye disease [22,23] as well as in the formulation of eye drops as viscosifying agent [24]. Furthermore, several works already demonstrated the suitability of HA-based nano-carriers in enhancing ocular drug delivery thanks to their interactions with superficial corneal components [23,25,26,27]. It was clearly evidenced that HA and functionalised-HA derivatives can increase the residence time of drugs on the corneal surface, thus decreasing drug drainage and improving drug bioavailability [25].

Taking into account all these aspects, both hydrophobic (dexamethasone (DEX) and piroxicam (PIR)) and hydrophilic (Tobramycin (TOB) and diclofenac (DCF)) drugs were loaded into self-assembled HA-cholesterol (HA-CH) NHs, and *ex vivo* transcorneal permeation experiments were performed to assess the capability of such NHs to promote the ocular delivery of these therapeutics, behaving as permeation enhancers. Grafting cholesterol (CH) moieties to the HA chains allowed the polymer to assemble spontaneously in aqueous environment thus forming NHs, composed by internal hydrophobic cholesterol domains interspersed within a hydrophilic environment of HA chains [28]. Such HA-CH-based NHs were able to efficiently entrap both hydrophobic and hydrophilic drugs [29,30]. Among the possible hydrophobic moieties, CH represents one of the best candidates, being biocompatible and occurring in cell membranes, including corneal epithelial cells [7,31]. Some compounds (e.g., cyclodextrins) were found to interact and extract cholesterol from the membrane of ocular epithelial cells, improving the corneal permeation of topical drugs [7]. Therefore, we believe that the CH moieties of HA-CH NHs may similarly interact with the epithelial cholesterol molecules via hydrophobic interactions, thus increasing the residence time of therapeutics on the cornea surface. To this end, both the interaction and the bioadhesive properties of NHs with an *ex vivo* cornea model were investigated.

## 2. Experimental Section

### 2.1. Materials

Hyaluronan tetrabutylammonium salt (HA^−^TBA^+^, molecular weight (M*_w_*) = 2.2 × 10^5^) was purchased from Hysilk (Dolní Dobrouč, Czech Republic). Cholesterol (CH), 4-bromobutyric acid, *N*-methyl-2-pyrrolidone (NMP), ethanol (EtOH), acetone, N-(3-dimethylaminopropyl)-N′-(ethylcarbodimide hydrochloride) (EDC∙HCl), dexamethasone (DEX), piroxicam (PIR), diclofenac sodium salt (DCF), Tobramycin (TOB), 4-(dimethylamino)pyridine (DMAP), phosphate buffered saline (PBS) tablets, O-phthalaldehyde (OPA), boric acid, 2-mercaptoethanol, isopentane and Haematoxylin and Eosin staining were purchased from Sigma-Aldrich (Milan, Italy).

### 2.2. Methods

#### 2.2.1. Synthesis of HA-CH and NHs Preparation 

The methods for the synthesis of the hyaluronan-cholesterol (HA-CH) derivative and the preparation of empty NHs formed by the self-assembling of HA-CH molecules were already described in detail in previous works [28,32].

#### 2.2.2. Synthesis of Fluorescent HA and NHs (Rhod-HA and Rhod-NHs)

Fluorescent HA or NHs were synthetised as previously reported [33]. Briefly, 1.5 mg/mL of HA or HA-CH were dispersed in double distilled water under magnetic stirring overnight at 25 °C. HA-CH sample was then autoclaved (121 °C for 20 min) to allow the self-assembled NHs formation. A rhodamine B-isothiocyanate (Rhod) stock solution in DMSO (9 mg/mL) was added to HA solution or NHs suspension (8 μL for 1 mg of polymers corresponding to a degree of functionalisation (DF) of 5.4 and 6.3% (% mol) for HA and HA-CH, respectively. The reaction mixtures were left for 5 h at 25 °C in the dark, exhaustively dialysed against distilled water and then freeze-dried. The final DF was assessed through UV–vis analysis: samples were solubilised in DMSO and checked at 550 nm at 25 °C. A Rhod calibration curve was obtained in the concentration range of 8.5–125 μg/mL in DMSO. DF was found to be 1 and 1.3% (mol of Rhod per mol of HA and HA-CH repeating units, respectively).

#### 2.2.3. Preparation and Characterisation of DEX or PIR-Loaded NHs

For the preparation of DEX or PIR-loaded NHs, samples of HA-CH (1 mg/mL, degree of functionalisation (Df) = 15%, mols of CH/mols of HA repeating units) were left under magnetic stirring in double distilled water overnight, at 25 °C. DEX or PIR were solubilised in acetone at the concentration of 2 mg/mL; 0.5 mL of each drug solution was allowed to evaporate by means of a Heidolph Hei-VAP rotary evaporator (Buchi, Schwabach, Germany) and the drug film was then added by 3 mL of HA-CH suspension, corresponding to a weight ratio of 0.33 (mg of DEX or PIR/mg of HA-CH). The mixtures were kept under magnetic stirring for 1.5 h at 25 °C and then autoclaved (121 °C, 1.1 bar for 20 min) to form DEX or PIR-loaded NHs. Samples were centrifuged at 4000 rpm for 10 min at 20 °C. Pellets (unloaded DEX or PIR) were used for the indirect quantification of entrapped DEX or PIR into NHs, whilst the supernatants (DEX/NHs or PIR/NHs) were analysed with a DLS equipment. 

#### 2.2.4. Preparation and Characterisation of TOB-Loaded NHs

Three milligrams of HA-CH (Df of 15%) was left under magnetic stirring in 2.9 mL of PBS (0.01 M, pH = 7.4) overnight at 25 °C. Then, 0.1 mL of TOB solution (10 mg/mL) (corresponding to a weight ratio of 0.33 mg of TOB/mg of HA-CH) was added and the mixture and autoclaved for 20 min at 121 °C, leading to TOB/NHs formation. TOB/NHs were purified from free antibiotics via SEC. SEC was performed using Econo-Pac chromatography columns (Bio-Rad, Segrate, Italy) packed with Bio-Gel P-10 (polyacrylamide with an exclusion limit range of 1.5–20 × 10^3^, Bio-Rad, Segrate, Italy). Specifically, 1 mL of TOB/NHs mixtures was loaded into SEC and eluted with 10 mL PBS at 25 °C. The unloaded TOB was freeze-dried and used for the indirect quantification of entrapped DCF into NHs, whilst TOB/NHs were analysed with DLS.

#### 2.2.5. Preparation and Characterisation of DCF-Loaded NHs

Three milligrams of HA-CH (Df of 15%) was left under magnetic stirring in 2.9 mL of PBS (0.01 M, pH = 7.4) overnight at 25 °C and then autoclaved (121 °C, 1.1 bar for 20 min) to form self-assembled NHs. Then, 0.1 mL of DCF solution (10 mg/mL) (corresponding to a weight ratio of 0.33 mg of DCF/mg of HA-CH) was added to NHs suspension and the mixture was left for 3 h under magnetic stirring at 25 °C. DCF/NHs were purified from free antibiotics via SEC. SEC was performed using Econo-Pac chromatography columns (Bio-Rad, Segrate, Italy) packed with Bio-Gel P-10 (polyacrylamide with an exclusion limit range of 1.5–20 × 10^3^, Bio-Rad, Segrate, Italy). Specifically, 1 mL of DCF/NHs mixtures was loaded into SEC and eluted with 10 mL PBS at 25 °C. The unloaded DCF was freeze-dried and used for the indirect quantification of entrapped DCF into NHs, whilst DCF/NHs were analysed with DLS. 

#### 2.2.6. Quantification of Entrapped DEX, PIR or DCF into NHs

DEX or PIR pellets and freeze-dried DCF (unloaded drugs) were solubilised in EtOH and quantified in order to obtain, by difference, the amount of entrapped drug into NHs. Analyses were performed by using a Knauer Azura HPLC instrument equipped with a binary pump (Azura P 6.1 L) and a UV-Vis detector (190–750 nm, Azura UVD 2.1 L), controlled by Clarity software. Samples (20 μL) were injected into a Knauer Eurospher II C18 column (5 μm, 4.6 × 250 mm); the samples were injected at 1 mL/min^−1^ in mixtures of water: acetonitrile 50:50 (isocratic mode) for DEX, in water (+ 0.1% *v/v* of TFA): acetonitrile (+ 0.1% *v/v* of TFA) 50:50 (isocratic mode) for PIR and in water (+ 0.1% *v/v* of TFA): acetonitrile (+ 0.1% *v/v* of TFA) (gradient mode) from 35:65 to 0:100 for DCF. The unloaded DEX was quantified at λ = 239 nm using a calibration curve previously recorded with DEX standard solutions in ethanol in the range 1.95–250 μg mL^−1^ (R^2^ = 0.999, *n* = 10); PIR and DCF were detected at λ = 356 nm and 276 nm, respectively, using a calibration curve in ethanol for PIR and in MeOH for DCF in the range 0.97–250 μg mL^−1^ (R^2^ = 0.999, *n* = 10).

Encapsulation Efficiency (*EE*) and drug loading (*DL*) of DEX/NHs, PIR/NHs and DCF/NHs were calculated by using the Equations (1) and (2):

(1) $$\%EE=\frac{concentration\;of\;loaded\;drug}{concentration\;of\;added\;drug}\times 100$$

(2) $$\%DL=\frac{concentration\;of\;loaded\;drug}{polymer\;concentration\;}\times 100$$

#### 2.2.7. Quantification of Entrapped TOB into NHs

The amount of unloaded TOB was quantified in order to obtain, by difference, the amount of the entrapped drug. As TOB does not absorb UV light, the aminoglycoside derivatisation with OPA was performed according to the literature [34]. For the preparation of OPA reagent, 618.3 mg of boric acid were dissolved in 100 mL of double distilled water and then the pH was adjusted to 9.7 with NaOH 1 M (final concentration 100 mM). Separately, 10 mg of OPA were dissolved in 1 mL of EtOH. Then, 100 µL of the OPA solution were added to 900 µL of sodium borate and 2 µL of 3-mercaptopropionic acid were added [35,36]. The unloaded TOB was freeze-dried to remove the solvent and solubilised in 200 µL of water: acetonitrile 50:50 mixture. Then, 40 µL of this mixture was added to 40 µL of OPA reagent (corresponding to 1:1 TOB:OPA volume ratio); the reaction was incubated for 30 min at room temperature and diluted 1:10 with water: acetonitrile 50:50 mixture for TOB quantification via HPLC. The sample was injected at 1 mL/min^−1^ in mixtures of acetate buffer (15 mM, pH = 6.5) and acetonitrile (gradient mode) from 20:80 to 0:100. A Symmetry C18 column (300 Å, 3 μm, 4.6 mm × 250 mm, 1/pk, Waters Corporation) was used for the separation. The fluorescence of the eluted sample (λ = 340/450) was analysed using a fluorescent detector (RF-551, Shimadzu) and the peak area was integrated by means of the Empower 2 software (Waters Corporation). The unloaded TOB was quantified using a calibration curve previously recorded with TOB standard solutions in water: acetonitrile 50:50-OPA reagent (corresponding to 1:1 TOB:OPA volume ratio) in the range 0.75–23.4 μg mL^−1^ (R^2^ = 0.999, *n* = 5).

EE and DL of TOB/NHs were calculated according to Equations (1) and (2).

#### 2.2.8. Dynamic Light Scattering (DLS) Measurements

Hydrodynamic diameter (Z-average size), size distribution and PDI of DEX/NHs, PIR/NHs, TOB/NHs and DCF/NHs were measured by DLS at 25 °C by using a Submicron Particle Sizer Auto Dilute Model 370 (NICOMP, Santa Barbara, CA, USA). The ζ-potential of drug-loaded NHs was measured by using a Zetasizer Nano ZS instrument (Model ZEN3690, Malvern Instruments) equipped with a solid state HeNe laser (λ = 633 nm) at a scattering angle of 173°. The electrophoretic mobility of the samples was converted in ζ-potential by using the Smoluchowski equation. For comparison, the hydrodynamic diameter and the PDI of empty NHs were also measured. 

#### 2.2.9. NHs Formulations for *Ex Vivo* Experiments

To obtain NHs formulations suitable for ophthalmic administration, the osmolarity and pH of the NHs suspensions were adjusted to 290 ± 10 mOsm/L and to pH 7.40 ± 0.05 by adding glycerol at the final concentration of 2.28 % *w/v* and the required volume of 0.1 M phosphate buffer to pH = 7.40. The osmolarity of the formulations was measured using a Knauer K7400 (Berlin, Germany) osmometer and the stability of NHs suspension at 4 °C in these conditions of osmolarity and pH was monitored by DLS for one week. 

#### 2.2.10. *Ex Vivo*, Corneal Permeation Studies with Rhod-NHs

Freshly excised porcine eyeballs were obtained from a local slaughterhouse and immersed in NaCl 0.9% *w/v*. Each cornea was carefully excised along with 2–4 mm of sclera tissue, washed three times with PBS and then clamped between the donor and acceptor chambers of a vertical Franz diffusion cell for corneal permeation experiments. The donor and acceptor chambers were filled with 5 and 1 mL of PBS buffer, respectively, and placed in a water bath at 37 °C to reach ~35 °C on the cornea surface. After 15 min, PBS was removed from donor chamber and replaced with 1 mL of Rhod-NHs or Rhod-HA (as control). After 0.5, 4 and 6 h of NHs incubation, corneal buttons (6 mm) were isolated, washed three times with fresh PBS and embedded in Optimum Cutting Temperature compound (Tissue-Tek OCT; Sakura) and then snap frozen in nitrogen-cooled isopentane (Sigma-Aldrich). Embedded tissues were frozen at −20 °C for 24 h and then stored at −80 °C. Transversal sections of 10 μm thickness were obtained by cryostat sectioning and placed on microscope slides (Thermo Scientific, Waltham, MA, USA). In order to evaluate Rhod-NHs fluorescence, sections were fixed in formalin solution and neutral buffered 10% (Sigma-Aldrich) for 10 min, and Hoechst staining was used to visualise nuclei. Fluorescence microscopy images were obtained using Axio Imager A2 microscope equipped with LED Colibri 7 for gentle fluorescence imaging (Carl Zeiss Microimaging, Inc., Dublin, CA, USA). To evaluate tissue integrity and general morphology, formalin fixed sections were stained with Haematoxylin and Eosin, according to standard protocols and photomicrographed using Axio Imager A2 (Carl Zeiss Microimaging, Inc.). Representative images were captured at the magnification of 10× and 20× by using Axiocam 503 colour (Carl Zeiss) and processed by ZEN2 software (Blue edition; Carl Zeiss). 

Furthermore, to investigate the ability of fluorescent NHs to cross the cornea, after 6 h of cornea incubation with Rhod-NHs, the buffer was removed from the receiving chamber of the Franz cell and analysed by using a UV-vis spectrometer (Perkin-Elmer double beam “Lambda 3A” model). Analyses were performed at 25 °C, using 10 mm quartz cuvettes (Hellma Analytics, Milan, Italy). Rhod-NHs aqueous solutions were detected in the range 800–300 nm.

#### 2.2.11. Bioadhesion Studies

The bioadhesive properties of NHs were investigated by DLS measurements, variable pressure scanning electron microscopy (VP-SEM) and fluorescence analyses. Mucin was hydrated with water by gentle stirring until complete dissolution to yield a 0.2% (*w/v*) dispersion at 25 °C.

##### Dynamic Light Scattering (DLS) Measurements

The bioadhesive interactions were studied by measuring the Z-average size and the ζ-potential of NHs suspension (1 mg/mL), mucin solutions at different concentrations (ranging from 0 to 1 mg/mL) and their mixtures (NHs: mucin ratios ranging from 0.01 to 1). All experiments were performed in triplicate (*n* = 3).

##### Variable Pressure Scanning Electron Microscopy (VP-SEM)

For VP-SEM studies, porcine corneas were incubated with NHs for 6 h in a vertical diffusion Franz cell, as described in Section 2.2.9. After 6 h, cornea samples were isolated, washed three times with fresh PBS and directly settled onto a carbon planchet stub. Surface observations of NHs on the outermost layer of the cornea in hydrated state without conductive coating were obtained using a variable pressure SEM (VP-SEM, Hitachi SU-3500). Experiments were performed at variable pressure combined with a cooling stage for limiting water vapour loss of the sample, during low-vacuum observations [37,38]. Images were recorded with the accelerating voltage of 8 kV, magnification of 1000× and working distance of 5.4 mm.

##### *Ex Vivo* Bioadhesion Assay

The retention of Rhod-NHs on freshly isolated porcine corneal tissue was also determined *ex vivo* using an experimental protocol previously described by Niamprem et al., with some modifications [39]. Briefly, corneal buttons (6 mm) were cut out with a trephine and held on a glass slide, and 50 μL of fluorescent NHs were instilled on the corneal surface. Next, the tissue was exposed to a continuous stream of simulated tear fluid (STF, pH 7.4; 35 °C) at a rate of 0.3 mL/min for 0, 5 and 10 min to induce shear stress mimicking blink action. Then, cryostat sections (10 μm) of the corneal tissue were prepared and imaged as described in Section 2.2.10. 

#### 2.2.12. *Ex Vivo*, Long-Term Corneal Permeation Studies with Drug-Loaded NHs

For the *ex vivo*, long-term corneal permeation studies, porcine corneas were excised as described in Section 2.2.9. Corneas were then washed three times with PBS and then held on a vertical Franz diffusion. The donor and acceptor chambers were filled with 5 and 1 mL of PBS buffer, respectively, and placed in a water bath at 37 °C to reach ~35 °C on the cornea surface. After 15 min, PBS was removed from donor chamber and replaced with 1 mL of DEX/NHs or PIR/NHs or TOB/NHs or DCF/NHs. Final drug amount in the donor chamber was equal to 184, 113 and 333 µg/mL for DEX/NHs, PIR/NHs and TOB/NHs and DCF/NHs, respectively. Each experiment was carried out for 6 h under gentle stirring. At scheduled time points (1, 2, 3, 4, 5 and 6 h), 1 mL was taken off from the acceptor chamber and replaced with 1 mL of fresh PBS, to maintain sink conditions. Each aliquot was freeze-dried and solubilised in 1 mL of EtOH (for DEX and PIR) or MeOH for (DCF). Samples were then injected into HPLC for quantifying the amount of permeated drug. After 24 h, each cornea was washed three times with 5 mL of PBS to remove, as much as possible, the free drug placed on its surface, cut in small pieces and left under magnetic stirring with 5 mL of EtOH (for DEX and PIR) or MeOH (for DCF), overnight at 4 °C to extract the drug entrapped into the cornea. Samples were then centrifuged at 4000 rpm for 10 min and the supernatant were analysed by using HPLC. As a control, free TOB and DCF solutions at the concentration of 333 µg/mL, were prepared and used in corneal permeation studies. All the experiments were performed in triplicate (*n* = 3).

#### 2.2.13. *Ex Vivo*, Short-Term Corneal Permeation Studies with DCF/NHs

For the *ex vivo*, short-term corneal permeation studies, porcine corneas were excised as described in Section 2.2.9. Each cornea was washed three times with TRIS-HCl (0.01 M, pH = 7.4) and then clamped between the donor and acceptor chambers of the Franz cell. The donor and acceptor chambers were filled with 5 and 1 mL of TRIS-HCl buffer, respectively, and placed in a water bath at 37 °C to reach ~35 °C on the cornea surface. After 15 min, TRIS-HCl was removed from donor chamber and replaced with 1 mL of DCF/NHs. Final drug amount in the donor chamber was equal to 333 µg/mL. Each experiment was carried out for 1.5 h under gentle stirring. At specific time points (15, 30, 45 min, 1, 1.25, and 1.5 h), 2 mL were withdrawal from the acceptor chamber and replaced with 2 mL of fresh buffer. Each aliquot was freeze-dried and solubilised in 100 µL of 0,1% formic acid (for DCF). Samples were then filtered with 0.22 µm regenerated cellulose membrane filters (Sartorius Italy s.r.l., Monza, Italy) and analysed to quantify the amount of permeated drug by using a Waters Acquity H-Class UPLC-MS (Waters, Milford, MA, USA) equipped with a quaternary solvent manager (QSM), a sample manager with a flow through needle system (FTN), a photodiode array detector (PDA) and a single-quadruple mass detector with electrospray ionisation source (QDa). Specifically, samples were injected onto a Kinetex C18 column (100 mm × 2.1 mm i.d., 2.6 μm particle size) and eluted in isocratic mode using a mobile phase consisting of 45% solvent A (0.1% formic acid in water) and 55% solvent B (0.1% formic acid in acetonitrile) at a flow rate of 0.5 mL min^−1^ and column temperature of 25 °C. The calibration curve was obtained by injecting increasing amounts of Diclofenac in the range 7.5–125 µg mL^−1^ (*R*^2^ = 0.98, *n* = 3). After 1.5 h, each cornea was washed three times with 5 mL of TRIS-HCl to remove the free drug placed on its surface, cut in small pieces and left under magnetic stirring with 5 mL of MeOH (for DCF), overnight at 4 °C to extract the drug entrapped into the cornea. Samples were then centrifuged at 4000 rpm for 10 min, and the supernatants were analysed by using HPLC. For a comparison, free DCF solution at the concentration of 333 µg/mL was also used in corneal permeation studies. All the experiments were performed in triplicate (*n* = 3).

#### 2.2.14. Statistical Analyses

For *ex vivo* permeation experiments, statistical significance was determined by using a two-way analysis of variance (ANOVA) with GraphPad Prism 5.0 Software (Graph Pad Software Inc., La Jolla, CA, USA). Differences between groups were determined by Tukey’s multiple comparison test. Asterisks denote statistically significant differences. Statistical significance was set to a *p*-value < 0.05.

## 3. Results

Topical application of ophthalmic drugs is pursued thanks to its ease and comfortable administration as well as patient compliance, even if it is affected by a low bioavailability, which considerably limits the efficacy of the drugs [19]. Hence, novel strategies are required. HA is a suitable material for the formulation of ocular drug delivery systems thanks to its bioadhesion properties as well as its CD44 binding capability [40]. We already developed amphiphilic HA-based NHs for drug delivery applications and loaded them with both hydrophobic and hydrophilic drugs [30,41]. Thus, this work is aimed at investigating the ability of self-assembled HA-CH NHs to adhere to the cornea surface and interact with its components similarly to HA, enhancing drug permeation through the cornea. Compared to other polysaccharide-based NHs intended for ocular drug delivery, the HA-CH NHs prepared for the present work show the further advantage of an easy-fast preparation procedure (sterile and drug-loaded NHs are formed in a single step by autoclaving process), which is amenable to their scale-up production as eye-drops.

### 3.1. Ex Vivo Transcorneal Permeation of Rhod-NHs 

Rhodamine B-isothiocyanate (Rhod) dye was covalently linked to the hydroxyl groups of NHs, and the obtained fluorescent NHs were employed for studying their ability to cross porcine corneas by using a Franz cell as well as their permeation pattern by fluorescence microscopy, after incubation at different time points.

Porcine corneas were used as they show biomechanical properties similar to those of human cornea as well a similar thickness, although small variations between central and peripheral locations are observed [12,42,43].

**Figure 1:**
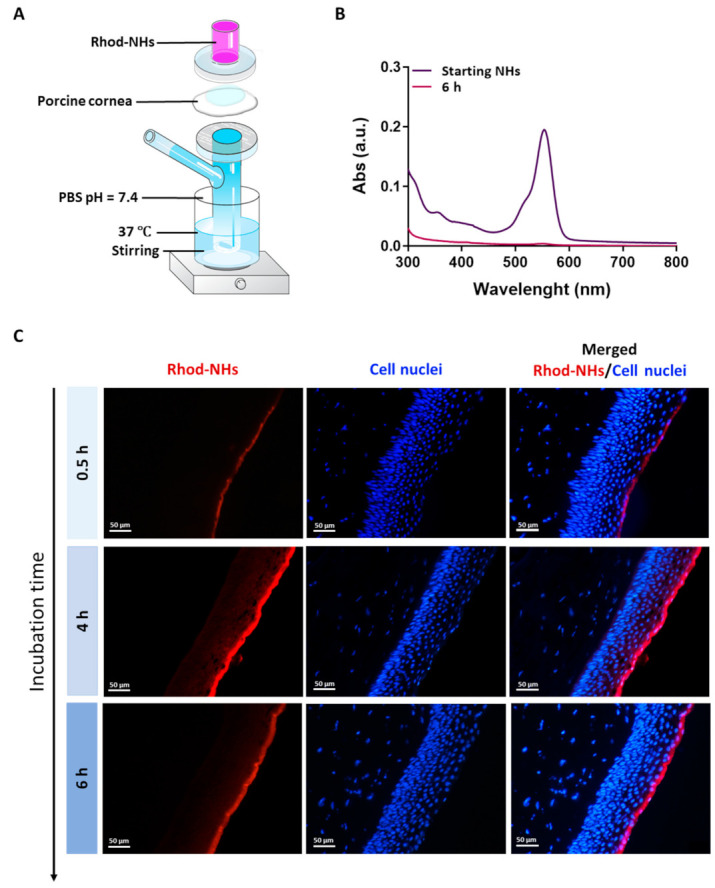
Experimental set-up used for the *ex vivo* transcorneal permeation experiments (**A**). UV absorbance of Rhod-NHs placed in the donor chamber of the Franz cell (dark purple line) and the solution in receiving chamber of Franz cell after 6 h of *ex vivo* transcorneal permeation experiment (purple line) (**B**). Fluorescence micrographs (scale bars: 50 μm) of vertical slices of porcine corneas after 30 min, 4 h and 6 h of incubation with Rhod-NHs (**C**).

All the samples were prepared in a Franz cell apparatus, Figure 1A. In these experiments, no fluorescence signal in the receiving chamber of the Franz cell after an incubation time of 6 h was detected (Figure 1B), hence suggesting that NHs were not able to permeate the whole porcine cornea.

Fluorescence images were recorded on porcine corneas after incubation with Rhod-NHs for 0.5, 4 and 6 h; negligible signal, due to the autofluorescence, was observed in control samples (data not shown). As shown from the qualitative evaluation of fluorescence (Figure 1C), the signal significantly increased over time. Specifically, after 0.5 h a weak, fluorescence signal was observed in the epithelium layer, whilst after 4 h and 6 h to Rhod-NHs exposure, a remarkable fluorescence was observed in deep epithelial layers, suggesting a great affinity of the Rhod-NHs for the ocular tissue.

**Figure A1:**
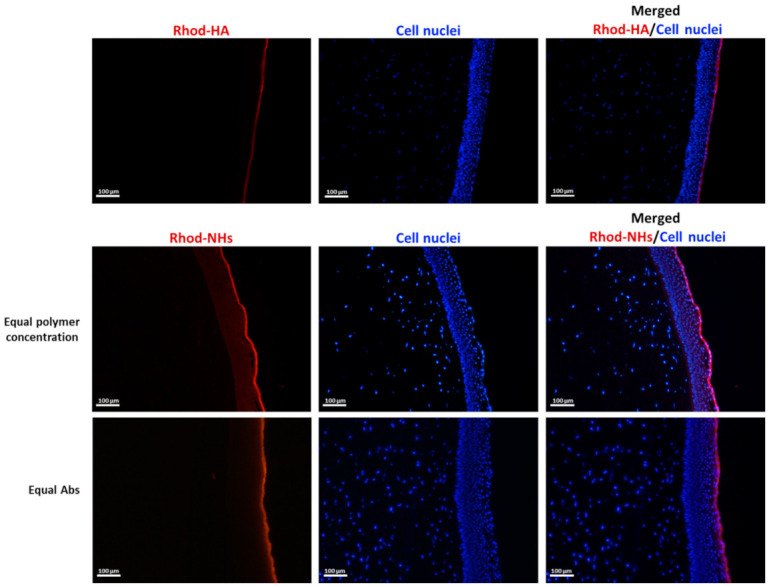
Fluorescence micrographs (scale bars: 100 μm) of vertical slices of porcine cornea after 4 h of incubation with Rhod-HA or Rhod-NHs.

As a control, also the fluorescence of porcine corneas after 6 h of incubation with Rhod-HA was investigated. Interestingly, a decrease in the fluorescence signal in the corneal epithelium was recorded for Rhod-HA (Figure A1), confirming a higher adhesion of HA-CH NHs to the corneal buttons in comparison to that of HA, probably due to the presence of hydrophobic interactions among the superficial corneal components and HA-CH NHs. Furthermore, the potential role of HA-based nanoparticles as penetration enhancers in ocular applications has been already reported [25]. 

However, Rhod-NHs were not able to penetrate the stroma even after an incubation time of 6 h, probably due to the densely packed collagen fibrils of the Bowman’s membrane beneath the epithelium, according to previous works [44,45]. 

### 3.2. Effect of HA-CH NHs on Corneal Integrity

**Figure 2:**
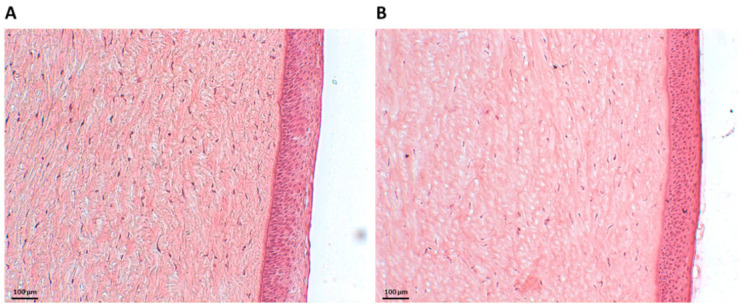
Histology of porcine corneal epithelium after incubation with NHs (**A**) or PBS (CTR) (**B**) for 6 h. Scale bars: 100 µm.

To assess the *ex vivo* biocompatibility of NHs, histology examinations were carried out. Figure 2A shows Haematoxylin and Eosin (H&E)-stained porcine corneal tissues after 6 h of incubation with NHs. For an appropriate comparison, H&E staining was also performed on porcine corneas treated with PBS for 6 h (Figure 2B). Tissue sections showed the corneal epithelium to be intact, with a neat and well-defined layered structure, thus confirming that NHs have no harmful effects on the cornea samples.

### 3.3. Bioadhesive Properties of NHs

**Figure 3:**
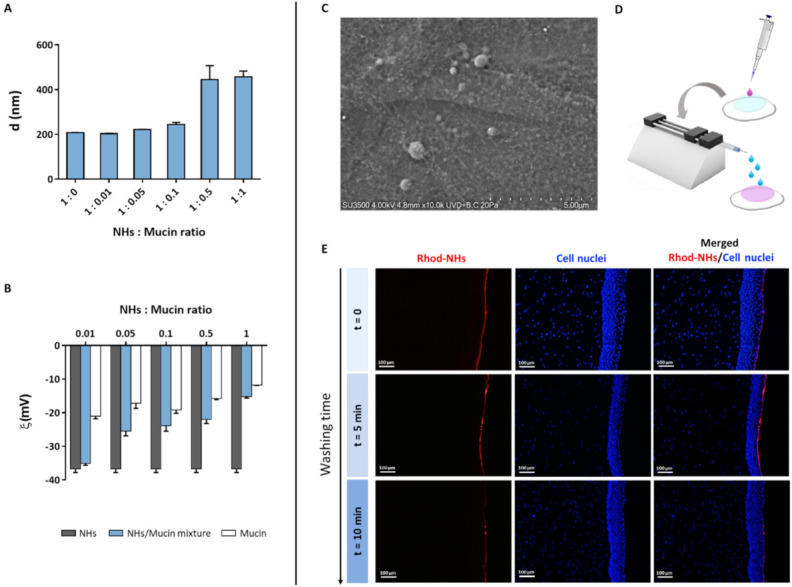
Mean diameter of NHs/mucin mixtures at different NHs: mucin ratio (**A**). ζ-pot of NHs, mucin and their mixtures at different NHs: mucin ratio (**B**). All data are expressed as the mean value ± standard deviation. Results were obtained in triplicate (*n* = 3). VP-SEM image of the porcine cornea after 6 h of incubation with NHs in its hydrated state (**C**). Schematic workflow for *ex vivo* bioadhesion studies with Rhod-NHs (**D**). Fluorescence micrographs (scale bars: 100 μm) of vertical slices of the porcine cornea after instillation of 50 μL of Rhod-NHs (0 min), followed by washing with STF (0.01 M, pH = 7.4) for 5 and 10 min (**E**).

As the tear fluid and blink action cause the drainage of ophthalmic drugs after their topical instillation, limiting their ocular retention on the cornea surface, polymeric carriers with bioadhesive properties have been widely used as ocular drug delivery systems [39]. Polymers (i.e., chitosan, HA) can interact with the corneal or conjunctival mucin via non-covalent bonds [46]. Therefore, to evaluate NHs bioadhesion, the mean diameter and ζ-pot of NHs with and without the addition of an increasing amount of mucin were measured (Figure 3A,B). The increase in mucin concentration (and hence in NHs: mucin ratio) leads to an increase in NHs size and a reduction in NHs ζ-pot. Such results suggest the formation of strong interactions between NHs and mucin, which depend on polymer−mucin ratio. Moreover, VP-SEM observations evidence the outermost layer of hydrated corneal epithelium, showing a thigh junction that joins the cell surfaces, wherein NHs adhere to the surface by interactions with the filamentous glycocalyx network covering epithelial cells and partially NHs (Figure 3C). Furthermore, the bioadhesive properties of HA-CH NHs were confirmed by fluorescence analyses. Specifically, 50 μL of Rhod-NHs were instilled on porcine corneas, followed by washing with STF for 5 and 10 min to mimic an eye drop application and the blink action and the tear flow, respectively (Figure 3D). As shown by Figure 3E, the fluorescence signal of corneal buttons after 5 min of washing, is similar to that of starting corneal samples (t = 0). Fluorescence intensity slightly decreases after 10 min of washing, even though a weak signal was still recorded, suggesting that NHs may be retained on porcine corneas and adhere to their surface. 

### 3.4. Preparation and Characterisation of DEX/NHs, PIR/NHs, TOB/NHs and DCF/NHs Formulations

**Figure 4:**
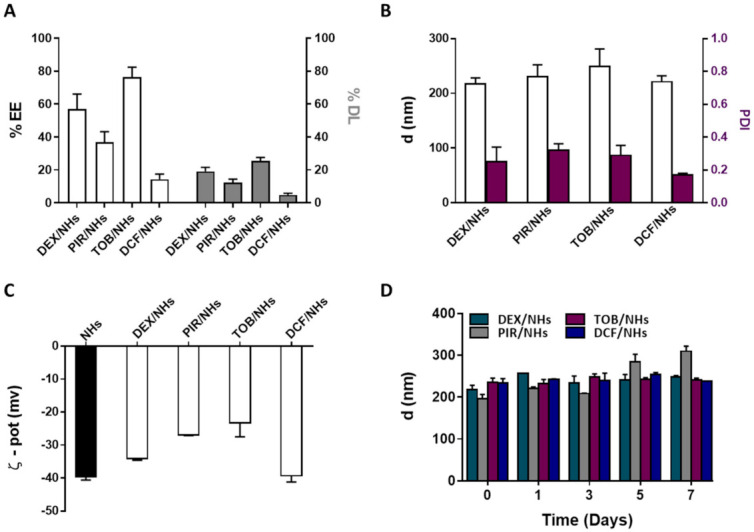
%EE and %DL of DEX/NHs, PIR/NHs, TOB/NHs and DCF/NHs (**A**). Mean diameter and PDI (**B**) and ζ-pot (**C**) of empty and drug-loaded NHs. Mean diameter of DEX/NHs, PIR/NHs, TOB/NHs and DCF/NHs in PBS (0.01 M, pH = 7.4) as a function of the time, at 4 °C (**D**). All data are expressed as the mean value ± standard deviation. Results were obtained in triplicate (*n* = 3).

DEX and PIR, respectively steroidal and non-steroidal anti-inflammatory drugs, poorly soluble in water, were loaded into NHs with the aim to enhance their water solubility as well as their transcorneal permeation to reach a high therapeutic efficacy. Both drugs were loaded into NHs by autoclaving and the obtained DEX/NHs and PIR/NHs were purified from the free drugs by applying a mild centrifugation (4000 rpm for 10 min). Similarly, TOB and DCF, which are hydrophilic anti-bacterial and anti-inflammatory drugs, respectively, were loaded into NHs to enhance their trans-corneal permeation, as the tight junctions of the corneal epithelium act as paracellular diffusion barriers for the diffusion of hydrophilic molecules [11]. Values of pH lower than 7 are not suitable for the formulation of DCF/NHs, leading to the formation of a precipitate immediately after the addition of the drug to the polymer suspension. For this reason, a different solvent (PBS 0.01 M, pH = 7.4 instead of double-distilled water, pH = 5) was used to prepare such drug-loaded NHs. TOB/NHs and DCF/NHs were purified from the unloaded drugs using a size exclusion chromatography (SEC). The %EE and %DL were quantified for all the NHs formulations by using HPLC. As shown in Figure 4A, %DL values were 19.1 ± 2.5 for DEX/NHs and 12.3 ± 2.1 for PIR/NHs, corresponding to NHs drug concentration of 184 and 114 µg/mL, respectively. As the water solubility is 89 µg/mL for DEX and 23 µg/mL for PIR, the entrapment of these drugs into NHs leads to an increase in their apparent water solubility of 2- and 5-fold, respectively. The good %DL and hence %EE values observed for both DEX/NHs and PIR/NHs can be ascribed to the formation of hydrophobic interactions between the cholesterol moieties of HA-CH and the poor water-soluble drugs. High %EE and %DL values were also observed for TOB/NHs (76.5 ± 6.0) thanks to the cationic nature of the antibiotic at the physiological pH, which establishes strong electrostatic interactions with the negatively charged HA chains. In contrast, DCF is negatively charged at physiological pH, leading to weaker interactions with HA-CH and, hence, to a very low %DL values (4.8 ± 1). All the drug-loaded NHs were characterised in terms of d_h_, PDI and ζ-pot, showing average sizes of 217 nm (DEX/NHs), 230 nm (PIR/NHs), 249 nm (TOB/NHs) and 221 nm (DCF/NHs); the sizes are similar for all the drug-loaded NHs with the exception of TOB probably because of its relatively high MW (Figure 4B). The ζ-pot net values of loaded-NHs (ranging from ≈ |23| to |39| mV) were similar to those of empty NHs (≈|40| mV) and high enough to ensure a good stability of the nano-formulations (Figure 4C). Ophthalmic formulations require a non-irritating osmolarity (<500 mOsm) as well as a pH value equivalent to that of the tear fluid (pH = 7.4). For this reason, both the pH and the osmolarity of DEX/NHs, PIR/NHs and TOB/NHs formulations were adjusted to 7.4 and 290 ± 10 mOsmol/L by addition of PBS and glycerol solution (20% *w/v*), respectively. For DCF/NHs mixture, only the osmolarity was adjusted to 290 ± 10 mOsmol with glycerol, as the system was formulated in PBS at pH 7.4. All tested formulations did not show differences in the mean diameter and PDI values for at least 7 days when stored at 4 °C, confirming the stability of their aqueous suspensions even after the addition of PBS and glycerol (Figure 4D). 

### 3.5. Ex Vivo Transcorneal Permeation Experiments of DEX/NHs, PIR/NHs, TOB/NHs and DCF/NHs Formulations

**Figure 5:**
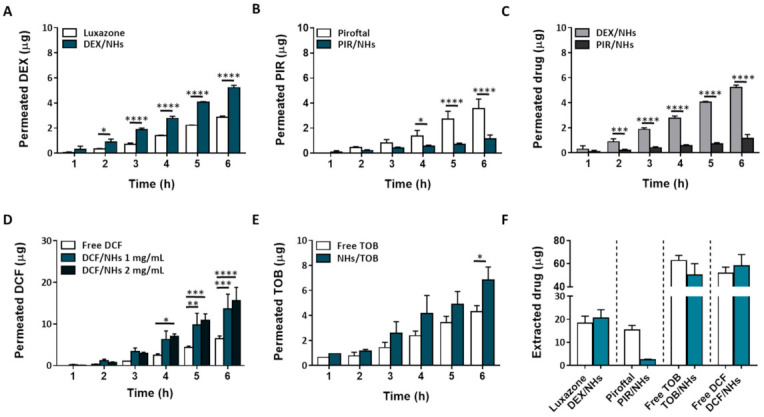
*Ex vivo* transcorneal permeation profiles for DEX/NHs and Luxazone (**A**), PIR/NHs and Piroftal (**B**), TOB/NHs and free TOB solution (**C**) and DCF/NHs and free DCF solution (**D**). Comparison of transcorneal permeation profiles of DEX/NHs and PIR/NHs (**E**). Corneal retention values of DEX, PIR, TOB and DCF comparing drug-loaded NHs and free drug solutions after 24 h of exposure (**F**). Asterisks denote statistically significant differences. The one-way analysis of variance (ANOVA) was carried out followed by Tukey’s multiple comparisons, and statistically significant differences were identified when *p*-values were lower than 0.05 (* *p* < 0.05), 0.01 (** *p* < 0.01), 0.001 (*** *p* < 0.001) and 0.0001 (**** *p* < 0.0001).

*Ex vivo* transcorneal permeation studies were performed for 6 h, as the histological investigation of porcine corneas confirms the integrity of the epithelial structure even after 6 h of NHs exposure. In Figure 5A–E, the transcorneal permeation profiles for both hydrophobic and hydrophilic drug-loaded NHs, as well as their controls, are reported. Specifically, free TOB and DCF solutions (at the same starting concentration of their NHs formulations) were used as TOB/NHs and DCF/NHs controls, respectively. On the other side, the aqueous suspensions of free DEX and PIR being not homogenous, their commercial formulations, Luxazone and Piroftal, were used as DEX/NHs and PIR/NHs controls, respectively. As shown by Figure 5A, DEX permeation through porcine corneas increases two times after 6 h as compared to Luxazone, suggesting that NHs may act as permeation enhancers increasing drug diffusion through the corneal tissue. Compared to DEX, the cumulative PIR amount permeated from PIR/NHs is significantly reduced (Figure 5C). According to fluorescence analyses, NHs are able to partially permeate the posterior epithelium of porcine corneas, thus increasing the drug amount that is able to reach the stroma. However, as stroma represents 90% of corneal thickness and is mainly made up of water, collagen, proteoglycans and keratocytes, it represents the rate-limiting barrier for the ocular delivery of hydrophobic drugs [47]. Consequently, even if HA-CH NHs may act as a permeation enhancer, increasing the diffusion of both the hydrophobic DEX and PIR through the epithelium, the amount of drug that crosses the whole cornea and reaches the receiving chamber of the Franz-cell is different and limited by the physical-chemical properties of the drugs, mainly the log P. Specifically, DEX (log P = 1.83) is more hydrophilic than PIR (log P = 3.06), with a DEX/PIR water solubility ratio of ~4. Thus, once the hydrophobic epithelium layer has been permeated, the amount of DEX that crosses the corneas depends on its water solubility, being ~four-folds higher than that of PIR. Similarly, the corneal retention of DEX after 6 h is also four-fold greater than that of PIR (Figure 5E). Furthermore, PIR permeation from PIR/NHs is also lower than that of its commercial formulation, Piroftal, in contrast with the results previously obtained for DEX. Such behaviour might be ascribed to the presence of viscosity-enhancing polymers in the commercial formulation (i.e., poly-vinyl pyrrolidone (PVP)) which improves the residence time of the formulation [48]. Furthermore, PVP is able to form complexes with bioactive molecules, thus facilitating the dissolution of many hydrophobic drugs (Piroftal is a solution, whilst Luxazone is a suspension) [49]. 

Furthermore, drug fluxes (J_s_) and permeability coefficients (K_p_) through porcine cornea were calculated and reported in Table 1. The values were calculated at the steady state per unit of area by linear regression analysis of permeation data, using the following equation:

J_s_ = Q (A ×·t)^−1^ (µg cm^−2^ h^−1^) (3)

where Q is the amount of drug permeated through the corneal barrier (µg) which reaches the acceptor chamber of the Franz cell; A is the active area available for permeation (0.6359 cm^2^) and t is the incubation time.

The permeability coefficient (K_p_) was calculated by applying the following equation:

K_p_ = J_s_ C_D_^−1^ (cm h^−1^) (4)

where C_D_ is the initial drug concentration loaded into the donor chamber (µg cm^−3^).

The results obtained with hydrophobic drugs suggest that HA-CH might be more effective in delivering hydrophilic therapeutics into the eye compared to hydrophobic drugs. Hence, to assess the capability of HA-CH NHs to facilitate the ocular delivery of hydrophilic drugs, the transcorneal permeation of DCF/NHs and TOB/NHs formulations was investigated. The K_p_ values of the DCF/NHs and TOB/NHs formulations were roughly twice those of the free drug solutions. Moreover, the J_s_ values of the DCF/NHs and TOB/NHs were 2.1-fold and 1.6-fold higher, respectively, than the J_s_ values of the free drug solutions. As expected, these results confirm that NHs significantly enhance both the permeation and the corneal retention of DCF and TOB as compared to the free drug solutions (Figure 5C,D).

**Figure 6:**
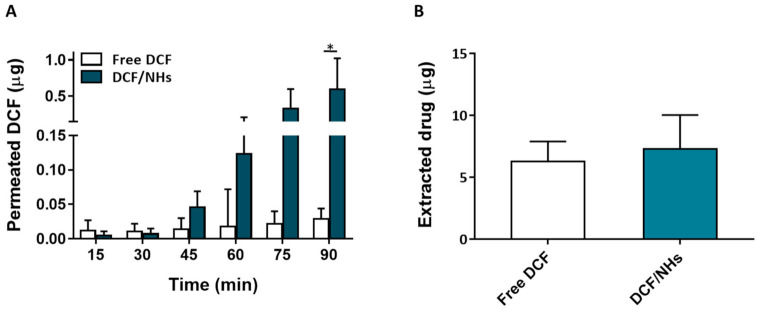
*Ex vivo* transcorneal permeation profile of DCF/NHs and free DCF (**A**). Corneal retention values of DCF comparing drug-loaded NHs and free drug solutions after 1.5 h of incubation (**B**). Asterisk denote statistically significant difference. The one-way analysis of variance (ANOVA) was carried out followed by Tukey’s multiple comparisons, and statistically significant differences were identified when *p*-values were lower than 0.05 (* *p* < 0.05).

For an eye drop application, the first minutes after the instillation of the formulation represent the most relevant time points. Therefore, *ex vivo* transcorneal permeation experiments of DCF, as NHs formulation or free drugs, were also performed for a very short incubation time point (1.5 h). As shown by Figure 6A, the corneal permeation of DCF from NHs formulation was similar to that observed for the controls (free drug solutions) after 30 min of exposure. After 45 min, the permeation of DCF from NHs significantly increases and becomes 20 times higher than their controls after 1.5 h. Furthermore, also the amount of the drug presents in cornea buttons after 1.5 h is greater for DCF/NHs than that of free DCF solutions. 

## 4. Discussion

Ocular drug delivery via topical instillation usually results in a limited therapeutic efficacy due to the anatomical ocular barriers [50]. Moreover, drug drainage due to the lachrymal fluid and the blinking action, as well as drug metabolism by lysosomal enzymes, are further complications for an ocular drug delivery [51]. Consequently, the use of nanocarriers with appropriate particle sizes and biocompatible and bioadhesive properties may represent an efficient strategy to topically treat several ocular disorders [5]. This work aims to demonstrate that NHs based on HA-CH are promising candidates for ophthalmic applications. HA, a biocompatible and biodegradable polysaccharide with bioadhesive properties, is already known for its implication in several ocular processes, as for the regeneration of corneal and conjunctival epithelial cells through interactions with its CD44 receptors [52].

In order to prepare HA-CH NHs, the carboxyl groups of HA were covalently linked to cholesterol moieties, leading to an amphiphilic HA-CH polymer, which is able to form self-assembled nano-sized structures after a suitable autoclave treatment (121 °C, 20 min) [32]. The use of autoclaving to prepare NHs shows several advantages: sterile and drug-loaded NHs (both with low molecular weight hydrophobic and hydrophilic drugs) are prepared in a single step with high reproducibility, although this approach is not suitable for thermo-sensitive drugs (i.e., DCF) [53]. Moreover, this strategy is also appealing for the scale-up production of such nanosystems. Previous works have already shown that HA-CH NHs may represent a useful drug delivery system in several fields of applications, thanks to their biocompatible and soft nature as well as their ability to efficiently entrap several therapeutics (i.e., small drugs, (poly)peptides) [54,55]. In the present work, hydrophobic DEX and PIR were successfully loaded into NHs, leading to a remarkable enhancement of their apparent water solubility and hence offering the opportunity to improve their bioavailability and therapeutic index (Figure 4A). The hydrophilic TOB was also successfully entrapped into NHs thanks to the formation of electrostatic interactions between the positively charged TOB and the negatively charged HA-CH NHs, opposite to what observed for the hydrophilic DCF, which is negatively charged at physiological pH (Figure 4A). The prepared nano-formulations showed a suitable range of sizes and appropriate values of PDI and ζ-pot for biomedical applications; they were also stable in simulated physiological conditions (pH and osmolarity) for at least one week (Figure 4B–D).

Lipid-based nanocarriers and NHs formed by bioadhesive polymers (e.g., chitosan, hyaluronic acid) have been shown to prolong the corneal residence time of ophthalmic drugs after topical instillation. The increased residence time offered by NHs avoids the frequent drug administration, thus improving patient compliance [56]. Puglia et al. prepared fluorescent-based solid lipid nanoparticles (LNs) to investigate their capability to interact with the ocular mucosa and to reach the posterior eye segment in in vivo rabbit models. Microscopy observation showed that the diffusion of such LNs begins at the corneal level in two hours, followed by their spreading to the back of the eye to reach the sclera and the retina after eight hours [57]. Moreover, polysaccharides can play a role in increasing the corneal residence time of the topically instilled drugs. Specifically, the bioadhesive properties of HA as well as its interaction with its receptors on the corneal epithelium may result in a prolonged precorneal-retention, as already reported in previous works [58,59]. To assess the bioadhesive properties of HA-CH NHs, both, *ex vivo* studies by fluorescence microscopy and in-tube analyses with mucin were performed (Figure 3A–E). Results show that HA-CH NHs are able to interact with corneal components, being retained on porcine corneas. Despite these bioadhesive properties, *ex vivo* studies showed that HA-CH NHs are not able to cross the corneal stroma, as confirmed by the red signal of Rhod-NHs, which was detected only in the epithelial layer, even after 6 h of incubation with NHs (Figure 1B). As this result is not in agreement with that previously observed by Puglia et al., further in vivo studies will be necessary to investigate the fate and the distribution of HA-CH NHs [57].

Furthermore, histology examination carried out on porcine corneas treated with HA-CH NHs showed the integrity of the corneal epithelium after 6 h of incubation with NHs, thus confirming that NHs have no harmful effects on the porcine corneas (Figure 2). These results clearly suggest that HA-CH NHs may be a favourable candidate for ocular drug delivery.

*Ex vivo* permeation experiments performed on porcine corneas with DEX-, PIR-, TOB- and DCF-based NHs formulations showed that HA-CH NHs play an active role in the permeation process of both hydrophobic and hydrophilic drugs through the porcine cornea. In particular, the permeation of the hydrophilic TOB and DCF is highly enhanced by NHs compared to the free drug solutions, whereas the permeation of hydrophobic DEX and PIR is strongly dependent on the water solubility of the entrapped molecules (Figure 5A–F and Figure 6A). These results confirm that NHs may act as permeation enhancers increasing drug diffusion through the corneal tissue. A similar permeation enhancer activity of amphiphilic HA derivatives was reported by Bongiovì et al., which ascribed this effect to the HA hydrophobisation [25]. Moreover, Liu et al., showed that hybrid polymer−lipid nanoparticles composed of chitosan and HA led to a higher corneal permeability of the antibiotic moxifloxacin hydrochloride compared to commercial formulations of the same drug, which might be due to the presence of chitosan and modified HA in their structure. Specifically, HA may promote the endocytosis of the nanoparticles thanks to its interactions with CD44 receptors [58]. Therefore, the permeation enhancer activity of HA-CH NHs in ocular drug delivery may be generally ascribed to both the interactions between the components of the corneal epithelium and the lipophilic CH moieties of the NHs and the interactions between HA and its CD44 receptor.

## 5. Conclusions

In this work, we have shown that HA-CH NHs can interact with superficial corneal components probably via hydrophobic interactions, enhancing their residence time on the corneal surface, and they can partially permeate the corneal epithelium. The prepared NHs can entrap both poorly water-soluble drugs, such as DEX and PIR, and hydrophilic drugs, such as TOB and DCF, leading to systems that showed small hydrodynamic diameters, suitable ζ-potential and PDI values and good stability under physiological conditions (pH 7.4, 290 mOsmol).

The entrapment of hydrophobic therapeutics into NHs results in different transcorneal drug permeation, which is limited by the water solubility of the drug. On the other hand, the delivery of hydrophilic drugs is highly enhanced by NHs, as evidenced by *ex vivo* transcorneal permeation experiments. Previous studies suggest the efficacy of HA-based materials for the treatment of several ocular diseases thanks to its excellent biocompatibility, biodegradability, bioadhesion and receptor interaction properties. Thus, the HA-CH NHs tested in the present work can represent an efficient and effective nano-carrier for delivering a wide range of bioactive molecules into the eye after topical administration, suitable for the medical treatment of several anterior and posterior segment ocular diseases.

## Figures and Tables

**Table 1:** Flux (J_S_) and permeability coefficient (K_P_) values, calculated from the porcine cornea permeation experiments, for DEX, PIR, DCF and TOB as free drug solution/suspension or drug-loaded NHs.

Drug	NHs		Control	
	J_s_ (µg cm^−2^ h^−1^)	K_p_ (cm h^−1^)	J_s_ (µg cm^−2^ h^−1^)	K_p_ (cm h^−1^)
DEX	1.367 ± 0.05	0.0072 ± 0.0003	1.216 ± 0.05	0.0040 ± 0.0002
PIR	0.3025 ± 0.05	0.0025 ± 0.0002	0.9349 ± 0.08	0.008 ± 0.0002
DCF	3.575 ± 0.18	0.0107 ± 0.0091	1.685 ± 0.11	0.0055 ± 0.0003
TOB	1.764 ± 0.08	0.0053 ± 0.0004	1.109 ± 0.09	0.003 ± 0.0001

## Data Availability

Not applicable.
